# One-step Hydrothermal Liquefaction and Catalytic Upgrading of Wastewater-Grown Microalgae for Potential Sustainable Aviation Fuel Precursors

**DOI:** 10.1021/acsomega.5c10732

**Published:** 2026-01-20

**Authors:** Bianca Barros Marangon, Jackeline de Siqueira Castro, Fabiane Carvalho Ballotin, Laís Santos Silva, Paula Assemany, Eduardo Aguiar do Couto, Thiago Abrantes Silva, Maurino Magno Jesus Junior, Vinícius José Ribeiro, José Ivo Ribeiro Júnior, Ana Márcia Carvalho, Sarah de Paiva Silva Pereira Pinheiro, Sergio Antonio A. Fernandes, Maria Lúcia Calijuri

**Affiliations:** † Department of Civil Engineering, Federal University of Viçosa (Universidade Federal de Viçosa), 36570-000 Viçosa, Minas Gerais, Brazil; ‡ Department of Chemistry, 67739 Federal University of Lavras (Universidade Federal de Lavras), 37200-000 Lavras, Minas Gerais, Brazil; § Department of Chemistry, 28120 Federal University of Viçosa (Universidade Federal de Viçosa), 36570-000 Viçosa, Minas Gerais, Brazil; ∥ Department of Environmental Engineering, Federal University of Lavras (Universidade Federal de Lavras), 37200-000 Lavras, Minas Gerais, Brazil; ⊥ Department of Soils, Federal University of Viçosa (Universidade Federal de Viçosa), 36570-000 Viçosa, Minas Gerais, Brazil; # Department of Statistics, Federal University of Viçosa (Universidade Federal de Viçosa), 36570-000 Viçosa, Minas Gerais, Brazil; ¶ Department of Forestry Engineering, Federal University of Viçosa (Universidade Federal de Viçosa), 36570-000 Viçosa, Minas Gerais, Brazil

## Abstract

In the context of aviation decarbonization goals, this study investigated the conversion of wastewater-grown microalgae into sustainable aviation fuel (SAF) precursor via one-step hydrothermal liquefaction (HTL) and upgrading. The highest bio-oil yield achieved was 23.07% (dry basis), at 320 °C for 30 min with 10% NiMo/Al_2_O_3_ catalyst. The highest heating value, 41.77 MJ kg^–1^, was obtained under the same temperature and catalyst concentration, but with a longer reaction time (120 min). Bio-oil yield was significantly influenced by temperature, while the catalyst played a key role in sulfur reduction. The response surface analysis identified an intersection region, around 324 °C with 15% NiMo/Al_2_O_3_ catalyst, that offers a favorable balance between high yield and low sulfur content. Since reaction time had no significant impact on the results, a 30 min process is recommended to improve energy efficiency. Bio-oil presented a predominance of aromatic hydrocarbons, with smaller fractions of alkanes and cycloalkanes, compounds that are desirable for SAF. These results highlight HTL as a promising pathway for wastewater-grown microalgae valorization. However, further refining and comprehensive technical, economic, and environmental assessments are needed to advance the use of this bio-oil as SAF precursor.

## Graphical abstract

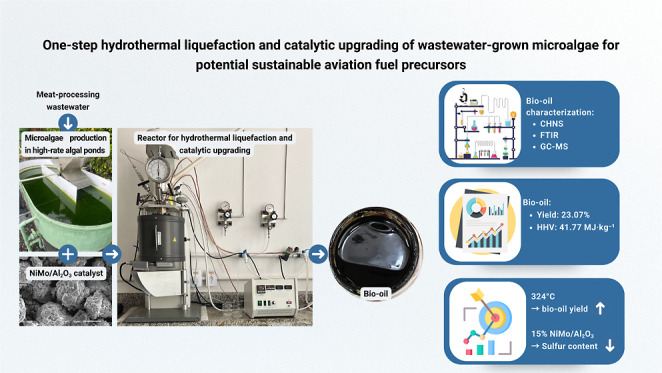

## 1 Introduction

The intensive use of fossil fuels and the growth of global air travel have intensified concerns regarding greenhouse gas (GHG) emissions. The aviation sector, which accounts for 2.5% of global carbon dioxide (CO_2_) emissions, has adopted sustainable aviation fuels (SAF) as a central strategy to achieve long-term decarbonization goals under international frameworks such as the Paris Agreement and the Carbon Offsetting and Reduction Scheme for International Aviation (CORSIA).^1–3^ However, existing SAF production pathways rely primarily on lipid-rich crops, waste oils, and lignocellulosic biomass, which face constraints related to cost, land availability, and competition with food production.^3^ These limitations have motivated the search for alternative feedstocks capable of providing both environmental and economic advantages.

Wastewater-grown microalgae have emerged as a promising SAF feedstock because they simultaneously enable biomass production, nutrient removal, and wastewater treatment and offering environmental cobenefits. In addition, microalgae cultivation does not require arable land and can use nonpotable water sources, avoiding competition with agriculture.^4–6^ Previous studies have highlighted the potential of wastewater-derived microalgae biomass for biofuel production via thermochemical routes, including hydrothermal liquefaction (HTL) and pyrolysis.^7,8^ Notably, microalgae grown in wastewater typically present higher ash content and more complex inorganic profiles than pure-culture strains, which affect conversion pathways, catalytic behavior, heteroatom distribution, and ultimately fuel quality.^5,6,8,9^ These characteristics underscore the importance of evaluating conversion technologies under realistic feedstock compositions rather than relying solely on pure-cultivated biomass.

Building on this context, previous work by Marangon et al.^10^ has provided important insights into the use of wastewater-grown microalgae for SAF-oriented thermochemical conversion. A previous study evaluated the technical challenges associated with HTL and catalytic hydrotreatment of wastewater-derived biomass, emphasizing issues such as heteroatom content, ash-induced catalyst deactivation, and limitations for meeting jet-fuel specifications.^10^ In a complementary assessment, Marangon et al.^11^ conducted a life-cycle analysis comparing different hydrothermal processing routes for wastewater-grown microalgae, demonstrating the environmental relevance and potential advantages of integrating biomass conversion with wastewater treatment systems. These studies established the technological and environmental foundations for the present work, while also highlighting the need for experimental optimization of catalytic HTL parameters for this type of feedstock.

HTL is one of the most promising routes for converting wet microalgae into bio-oil because it eliminates the need for energy-intensive drying steps and allows the production of a bio-oil containing hydrocarbons, phenols, ketones, esters, heterocycles, and nitrogenated compounds.^7,12–14^ Existing reviews have emphasized the relevance of HTL for SAF precursor production, the influence of reaction parameters, and the remaining technological challenges, including heteroatom removal and process integration.^13–17^ Following HTL, catalytic upgrading, typically via hydrotreatment using catalysts such as NiMo/Al_2_O_3_, is required to reduce oxygen, nitrogen, and sulfur contents while increasing the H/C ratio and improving the higher heating value (HHV) of the bio-oil. Previous studies have consistently shown that NiMo/Al_2_O_3_ promotes hydrodeoxygenation, hydrodenitrogenation, hydrodesulfurization, and aromatics hydrogenation in microalgae-derived biocrude, leading to higher carbon and hydrogen contents and a narrower hydrocarbon distribution compatible with jet-fuel precursors.^15,18–20^ Although upgraded bio-oil may contain hydrocarbons within the jet-fuel distillation range, further refining is generally required to meet ASTM D7566 specifications for SAF.^21^

Although HTL and upgrading of microalgae bio-oil have been widely investigated,^12,15,17,21–28^ studies specifically focused on wastewater-grown biomass are less reported.^7,8,10,29–31^ Those studies that do address wastewater-grown microalgae describe distinct conversion behaviors, including lower bio-oil yields, higher heteroatom content, and increased catalyst deactivation due to ash and inorganic constituents.^8,9,32^ This gap reinforces the need for integrated HTL and catalytic upgrading studies using biomass grown under uncontrolled, open-environment conditions, rather than pure cultures produced under tightly controlled laboratory settings. Previous works on one-step catalytic HTL have examined factors such as temperature, reaction time, and catalyst form.^22,23^ however, these studies generally employed fixed catalyst loadings and relied on pure-culture microalgae. Consequently, the influence of catalyst concentration on bio-oil yield and heteroatom removal for wastewater-derived microalgae has yet to be systematically evaluated.

To address these gaps, this study investigated the one-step HTL and catalytic upgrading of wastewater-grown microalgae, with a focus on understanding how temperature, reaction time, and catalyst concentration influence bio-oil yield, composition, and heteroatom removal. This biomass presented a distinct biochemical and inorganic profile compared to pure cultures, offering a more realistic scenario for biomass-to-SAF conversion. By clarifying the effects of key operational variables on hydrocarbon distribution, nitrogen and oxygen reduction, and fuel-relevant properties, the study aimed to provide experimental insights necessary for future integration with wastewater treatment systems and for techno-economic and environmental assessments of catalytic HTL routes.

To achieve this, a response surface methodology (RSM) with a central composite design was applied to systematically evaluate the influence of temperature, reaction time, and catalyst loading on the performance of the one-step process. The specific objectives were: (i) to assess the conversion of wastewater-grown microalgae into bio-oil through integrated HTL and catalytic upgrading, and (ii) to identify optimal operational conditions that maximize bio-oil yield while improving fuel quality for potential SAF precursor pathways.

## 2 Material and Methods

### 2.1 Biomass Production and Characterization

The microalgae biomass was obtained during the treatment of wastewater from a meat-processing industry using high-rate algal ponds (HRAPs) with working volume of 1 m^3^. The primary activity of this industry is the production of processed meat products derived from poultry, swine, cattle, and fish (such as mortadella, sausages, deli meats, smoked chicken breast, and shredded codfish). The wastewater used in this study originated from various production stages and from cleaning floors and equipment and was collected after the flotation system (primary effluent). The wastewater used for microalgae cultivation presented moderate organic load and suspended solids, with total suspended solids (TSS), ammoniacal nitrogen (N–NH_3_), soluble phosphorus, and TOC values consistent with typical meat-processing effluents. A detailed characterization of the wastewater composition is provided in Table S1 (Supporting Information).

The HRAPs were operated until the algae growth decay phase was reached, monitored through chlorophyll-a levels. After this period, paddlewheel rotation was stopped, and the biomass was collected via gravitational sedimentation.

The biomass was characterized in terms of phytoplankton community, biochemical composition, proximate and ultimate analysis, as described in Table S2 (Supporting Information). Table 1 presents the dominant phytoplankton species identified in the microalgae community and the biomass biochemical composition (lipids, proteins, and carbohydrates), proximate analysis (ash, volatile matter, fixed carbon, and moisture), and elemental composition (C, H, N, S, and O). Carbohydrates and oxygen were determined by difference. These data provide a comprehensive overview of the biomass composition used as feedstock in the one-step hydrothermal liquefaction and catalytic upgrading experiments.

**Table 1:** Characterization of the Wastewater-Grown Microalgae Biomass Used in This Study^a^

organisms	relative abundance (%)
chlorophyceae	
*Chlorella vulgaris*	23
*Scenedesmus acunae*	1
*Tetradesmus obliquus* (*Scenedesmus obliquus*)	72
Bacillariophyceae	
*Nitzchia* sp	4

biochemical composition	(wt %, dry basis)
total lipids	16.10
proteins	34.77
carbohydrates	24.12

proximate analysis	(wt %, dry basis)
ash	18.55
volatile matter	70.65
fixed carbon	4.34
moisture	6.46

elemental analysis	(wt %, dry basis)
C	41.40
H	6.48
N	6.66
S	0.74
O	26.17

a Note: analytical methods: lipids—Soxhlet extraction (AOAC, 2000); proteins—Kjeldahl method (APHA et al., 2023); carbohydrates—by difference (Wang et al., 2018); proximate analysis—ASTM D3172 (ASTM, 2021); CHNS—Elementar Vario Micro Cube analyzer; O—by difference.

### 2.2 Experimental Design and Statistical Analysis

Lower and upper limits for the operational conditions of HTL and one-step upgrading were selected based on temperature, reaction time, and catalyst proportion values that could accommodate both reactions, being:

- **270 °C ≤ Temperature ≤ 370 °C**—this range encompasses the temperature of the best bio-oil yield in one-step HTL and upgrading with NiMo/Al_2_O_3_ found by Moazezi et al.^23^ (287 °C) and is under subcritical water point conditions (<374 °C, 221 bar).^13^
- **30 min ≤ Time ≤ 120 min**—this range encompasses the time of the highest bio-oil yield in one-step HTL and upgrading with NiMo/Al_2_O_3_ obtained by Moazezi et al.^23^ (40 min) and a value commonly used for microalgae bio-oil upgrading (120 min).^21,24,25^

- **0% ≤ Catalyst ≤ 20%**—this this range encompasses the NiMo/Al_2_O_3_ concentration where Moazezi et al.^23^ obtained the highest heteroatoms removal (5%).

To achieve the expected results, the experimental design was carried out using Minitab 17 (trial version), following a Face-Centered Central Composite Design (FCCCD) (Table S3, Supporting Information), with triplicates at the central point and six axial points, totaling 17 reactions (Table 2). The FCCCD was adopted due to its suitability to the defined operational limits of temperature, time, and catalyst percentage, practical feasibility, and experimental safety, while also supporting a robust statistical design with reduced experimental demand. Table 2 summarizes the operational conditions of the one-step hydrothermal liquefaction and catalytic upgrading experiments, including the combinations of temperature (270–370 °C), reaction time (30–120 min), and NiMo/Al_2_O_3_ catalyst concentration (0–20 wt %) tested in each batch.

**Table 2:** Operational Conditions for the One-step Hydrothermal Liquefaction and Catalytic Upgrading Experiments

batch ID	experimental condition		
	temperature (°C)	time (min)	catalyst (%)
1	270	30	0
2	270	120	0
3	270	75	10
4	270	30	20
5	270	120	20
6	320	75	0
7	320	30	10
8	320	75	10
9	320	75	10
10	320	75	10
11	320	120	10
12	320	75	20
13	370	120	0
14	370	30	0
15	370	75	10
16	370	30	20
17	370	120	20

Based on the bio-oil yield and its elemental composition (CHNSO), the second-order Response Surface Model was adjusted as a function of the tested parameters.

### 2.3 One-step HTL and Catalytic Upgrading

The experiments were carried out in a 0.250 L stainless steel Parr batch reactor, model 4576, equipped with a model 4848 controller unit (Parr Instruments, IL, USA), a PID-controlled electric heating furnace, an adjustable stirrer (set to ∼150 rpm during the experiment), a pressure gauge, and a type J thermocouple to monitor the temperature inside the reactor.

For each batch, 12 g of freeze-dried biomass and 120 mL of distilled water were loaded into the reactor, resulting in a biomass-to-water ratio of 1:10. Before heating, the reactor was purged with hydrogen (H_2_) for 5 min to remove air and establish a reducing atmosphere. Previous studies involving NiMo/Al_2_O_3_ catalysts report an initial inert gas purge (e.g., N_2_), followed by H_2_ introduction.^15,22,23^ However, in this work, direct H_2_ purging was adopted under controlled laboratory conditions to ensure O_2_ removal and to maintain reducing conditions from the onset of the reaction.

Furthermore, according to the experimental design, NiMo/Al_2_O_3_ catalyst was added at proportions of 0%, 10%, and 20%. The NiMo/Al_2_O_3_ catalyst was synthesized and characterized as described in the Supporting Information. Catalyst characterization included X-ray diffraction (XRD) to determine crystalline phases, Brunauer–Emmett–Teller (BET) surface area analysis, and scanning electron microscopy coupled with energy-dispersive X-ray spectroscopy (SEM–EDS) for surface morphology and elemental composition. These analyses confirmed the expected crystalline structure, dispersion of active metals, and textural properties consistent with NiMo/Al_2_O_3_ catalysts reported in the literature (Figures S1–S3).

After the desired reaction time, the reactor heating was turned off and the heating jacket was removed. Cooling was performed using the built-in water-cooling coil system, which circulates tap water through the reactor jacket to facilitate heat removal and allow a gradual return to ambient temperature and pressure before product collection.

### 2.4 Products Separation

Figure 1 illustrates the separation procedure used to obtain the four products of HTL: gas phase, aqueous phase (water-soluble compounds), solid residues, and bio-oil. This procedure was adapted from Silva et al.^8^

**Figure 1:**
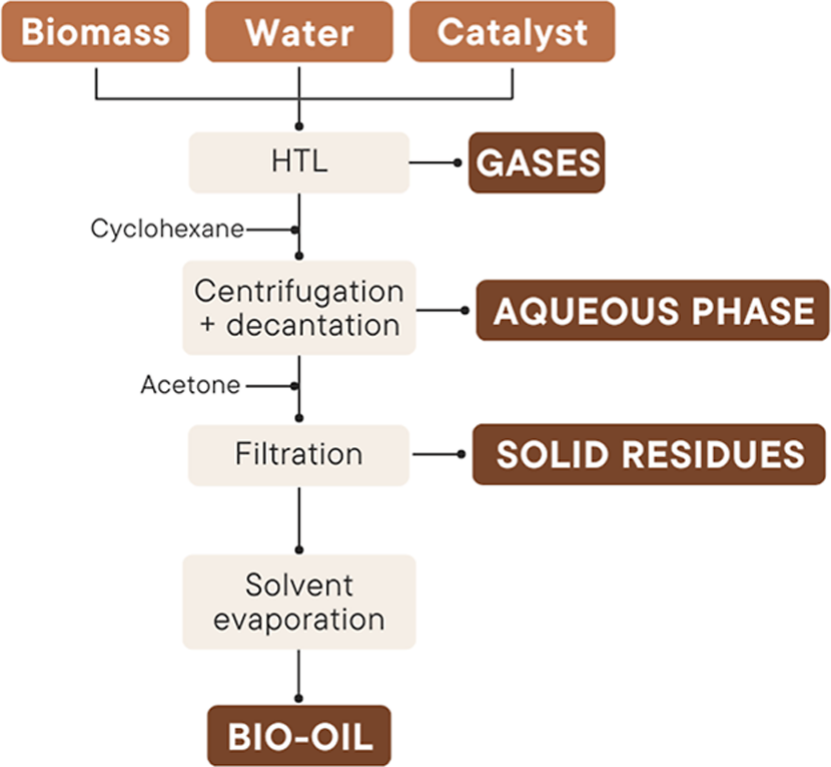
Separation procedure of HTL products.

After the reactor was cooled, the gas phase was released, and its volume was measured using a DAEFLEX G4 gas meter. Then, 1000 μL of gas were collected for analysis using insulin syringes equipped with Teflon push-button valves (Supelco Analytical—Sigma-Aldrich, Bellefonte, PA, USA).

After opening the reactor, cyclohexane was added (2 mL per gram of biomass) to facilitate the extraction of hydrophobic organic compounds and improve the separation between the bio-oil and aqueous phases. Although phase separation can occur by gravity settling, the addition of cyclohexane, used in other studies,^8,33^ enhances the recovery of partially soluble intermediates and reduces bio-oil losses. The reactor contents were then centrifuged at 3500 rpm for 10 min, yielding a supernatant (containing bio-oil, aqueous phase, and cyclohexane) and a sediment (bio-oil mixed with solid residues).

The supernatant was transferred to a separation funnel and allowed to settle for 30 min. The aqueous phase was then transferred to a preweighed rotary evaporator flask. After complete evaporation of the water, the flask was weighed again to determine the dry yield of water-soluble products.

The sediment from the centrifuge tubes and the supernatant from the separation funnel were washed using acetone as a solvent and filtered through a preweighed nylon membrane filter (47 mm diameter, 0.22 μm pore size). The membrane filter and solids were dried at 40 °C for 24 h and then weighed at room temperature to determine the yield of solid residues.

Bio-oil was separated from the solvents by N_2_ purging at a rate of 1 L min^–1^ for 6 h in a preweighed impinger. After approximately 18 h in a desiccator (to stabilize the impinger weight), it was weighed to determine the bio-oil yield.

### 2.5 Bio-oil Characterization

The carbon, hydrogen, nitrogen, and sulfur (CHNS) content of the bio-oil was determined using a Vario Micro Cube Elemental Analyzer. Helium and oxygen were used as the carrier and ignition gases, respectively. Bio-oil samples of 2 mg were stored in capsules and fully incinerated at 1200 °C. Oxygen content was calculated by difference. The HHV of the bio-oil was estimated according to Perry and Chilton.^34^ The atomic H/C and O/C ratios were calculated by dividing the percentage of each element (H, O, N, S) by the percentage of C and adjusting for their respective atomic mass ratios.

The chemical composition of the bio-oil was analyzed by gas chromatography coupled with mass spectrometry (GC–MS), using a SHIMADZU GCMS-QP2010 Ultra system. The interface temperature was set to 290 °C. The column used was a SPB-5 (30 m × 0.25 mm × 0.25 μm) (Supelco). The chromatographic conditions were as follows: injector temperature of 300 °C and detector temperature of 200 °C; the initial column temperature was 80 °C, held for 2 min, followed by a heating rate of 8 °C min^–1^ until reaching 140 °C, then a second heating rate of 4 °C min^–1^ until reaching 280 °C, which was maintained for 2 min. Helium was used as the carrier gas at a flow rate of 0.82 mL min^–1^, with a split ratio of 1:10. Compound peaks were identified based on mass spectral data libraries NIST 11 and 11s.

Organic functional groups contained in the biomass and bio-oil samples were recorded by Fourier-transform infrared spectroscopy (FTIR) using a spectrophotometer (ALPHAA II, BRUKER, USA) equipped with an attenuated total reflectance (ATR) accessory over the range of 400–4000 cm^–1^ with 64 scans and 4 cm^–1^ spectral resolution.

### 2.6 Byproducts Characterization

#### 2.6.1 Aqueous Phase

The identification and quantification of water-soluble compounds (aqueous phase) were carried out by high-performance liquid chromatography (HPLC) on a SHIMADZU chromatograph (SHIMADZU, SP, Brazil) coupled with a refractive index detector (RID), model RID-20A. Concentrated sulfuric acid (H_2_SO_4_) was added to the aqueous phase samples, which were then frozen. For HPLC analysis, an HPX 87H column (Aminex) (300 mm × 7.8 mm) and a guard column of the same phase (Bio-Rad Lab, RJ, Brazil) were used, with a flow rate of 0.7 mL min^–1^, a runtime of 25 min, and an oven temperature of 45 °C. The injection volume was 10 μL, and the mobile phase was acidified water (0.005 M H_2_SO_4_). Data were obtained using Lab Solutions software, Shimadzu Corporation (2013), according to standard curves for acetic, propionic, and butyric acids (Sigma-Aldrich, St. Louis, MO, USA). Formic, acetic, propionic, citric, lactic, butyric, isobutyric, valeric, isovaleric, and crotonic acids were analyzed.

#### 2.6.2 Solid Phase

Solid residues were characterized for CHN content using a PerkinElmer Series II 2400 Elemental Analyzer.

#### 2.6.3 Gaseous Phase

The gas phase was analyzed using a Shimadzu Nexis GC-2030 gas chromatograph equipped with a thermal conductivity detector (TCD). The detection of CO_2_, N_2_, H_2_, and CH_4_ levels was carried out using a Carboxen 1010 PLOT column (30 m × 0.53 mm), with argon as the carrier gas.

## 3 Results and Discussion

### 3.1 Bio-oil Yield and Elemental Composition

Table 3 presents the bio-oil yield and higher heating value (HHV) obtained under the different reaction conditions. Bio-oil yield was calculated as the ratio between the dry mass of bio-oil and the dry mass of microalgae biomass used in each reaction. The HHV was estimated from the elemental composition according to Perry and Chilton.^34^ Standard deviation values are reported for the central point replicates. Replicates were performed only at the central point to assess model reproducibility, following standard FCCCD methodology. The detailed CHNSO composition data are provided in the Supporting Information (Table S4).

**Table 3:** Bio-oil Yield and Higher Heating Value (HHV) Obtained from the One-step Hydrothermal Liquefaction and Catalytic Upgrading Experiments

batch ID	experimental condition			bio-oil yield (wt %, dry basis)	HHV (MJ kg^–1^)
	temperature (°C)	time (min)	catalyst (wt %)		
1	270	30	0	12.62	36.87
2	270	120	0	17.47	39.17
3	270	75	10	16.31	33.10
4	270	30	20	17.87	35.68
5	270	120	20	17.86	38.00
6	320	75	0	22.77	37.55
7	320	30	10	23.07	38.61
8 (center point)	320	75	10	21.52 ± 0.95	38.00 ± 1.05
9	320	75	10	22.50	39.18
10	320	75	10	20.60	37.61
11	320	120	10	22.01	41.77
12	320	75	20	20.87	39.88
13	370	120	0	20.38	38.20
14	370	30	0	19.38	36.39
15	370	75	10	19.14	38.40
16	370	30	20	18.33	38.14
17	370	120	20	17.47	38.78

The highest bio-oil yield (23.07 wt %, dry basis, or 28.78 wt %, ash-free basis) was obtained at 320 °C for 30 min with 10 wt % NiMo/Al_2_O_3_ catalyst, while the lowest (12.62 wt %, dry basis) occurred at 270 °C for 30 min without catalyst. On average, 73.17 wt % of the biomass was converted into HTL products under the tested conditions.

The superior yield at 320 °C is likely related to the optimal balance between depolymerization and secondary recondensation reactions. At this moderate temperature, the breakdown of lipids and proteins is favored, while gas and char formation remain limited. The NiMo/Al_2_O_3_ catalyst may have further promoted mild hydrodeoxygenation, facilitating conversion of intermediates into bio-oil. Similar yields were reported by Silva et al.^8^ for wastewater-grown microalgae, whereas higher values were observed by Moazezi et al.^23^ using pure *Chlorella vulgaris* under catalytic conditions. Differences among studies are mainly attributed to biomass composition, ash content, and operational parameters. Overall, the results are consistent with previously reported trends for HTL of microalgae and confirm that moderate temperatures (≈300–330 °C) favor maximum bio-oil production.^8,33^

The HHV of the bio-oils ranged from 33.10 to 41.77 MJ kg^–1^, values comparable to those reported for microalgae-derived bio-oil in previous studies.^8,22,23,28,30^ This trend suggests that catalytic upgrading under moderate to high temperatures enhances bio-oil energy density and quality, aligning with results from similar NiMo/Al_2_O_3_-assisted HTL processes reported in the literature.^22,35–37^

### 3.2 Response Surface Analysis

Among the response variables tested (bio-oil yield and C, H, N, S and O contents), bio-oil yield and S content were the only significantly explained response variables by the HTL operational parameters, as shown in Table S5, in the Supporting Information. The bio-oil yield and S content showed significant dependence on temperature and catalyst concentration (*p*-value < 0.005). Bio-oil yield exhibited a quadratic relationship with temperature and a significant interaction with catalyst (*p*-value < 0.005), while S content depended quadratically on both factors (*p*-value < 0.005), with no interaction between them.

Temperature was also highlighted as a significant parameter for bio-oil yield in as Audu et al.,^29^ Basar et al.,^16^ and Moazezi et al.^23^ researches. The catalyst played a significant role, reinforcing its selective action in promoting desulfurization. According to the adjusted statistical models, the maximum bio-oil yield is estimated near 334 °C with no catalyst, while the minimum S content is predicted around 316 °C with approximately 18% NiMo/Al_2_O_3_ catalyst. As the optimal conditions for each response do not fully coincide, the combined response surface analysis highlights a region of intersection between both optimal zones, around 324 °C with 15% NiMo/Al_2_O_3_ catalyst, where a favorable balance between high bio-oil yield and low S content can be achieved (Figure 2).

**Figure 2:**
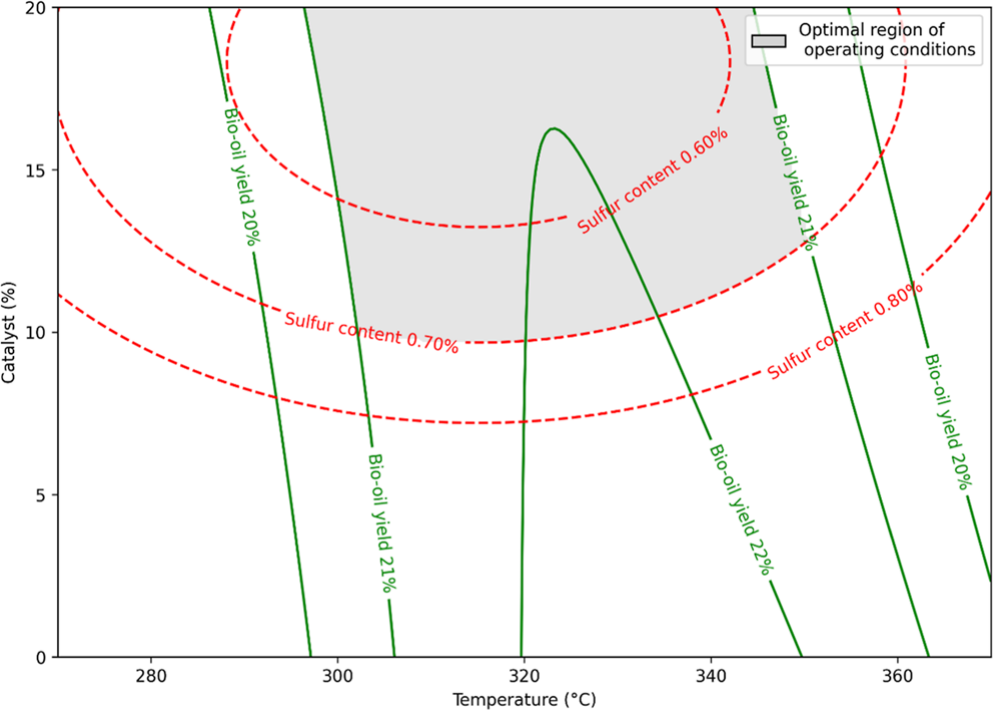
Effect of temperature, reaction time, and catalyst loading on bio-oil yield obtained from the one-step hydrothermal liquefaction and catalytic upgrading of wastewater-grown microalgae. Regression equations: Bio-oil yield (%) = −166.4 + 1.128 × *t* + 0.766*c* – 0.001685 × *t* ^2^ – 0.002400 × *tc* (*R* ^2^ = 84.46%); sulfur content (%) = 8.48 – 0.0460*t* – 0.0749 × *c* + 0.000073 × *t* ^2^ + 0002043 × *c* ^2^ (*R* ^2^ = 86.70%); * = significant by Student’s *t*-test at 5% of significance; *t* = temperature (270 °C ≤ *t* ≤ 370 °C) and c = catalyst (0% ≤ *c* ≤ 20%).

In the one-step HTL and upgrading process studied by the use of NiMo/Al_2_O_3_ catalyst was effective in reducing oxygenated and nitrogenous compounds and completely eliminating S compounds. According to Resurreccion and Kumar,^15^ catalysts like NiMo/Al_2_O_3_ have the potential to promote deoxygenation, hydrogenation, desulfurization, and denitrogenation reactions. Therefore, the use of the catalyst was effective in reducing the S content of the bio-oil while maintaining bio-oil yield values at optimal temperatures, partially fulfilling the objective of enhancing the bio-oil’s quality and productivity through catalytic HTL. Thus, further studies are still needed to improve this result, since there was a reduction in the contents of other heteroatoms (N and O), although not statistically related to the catalyst.

The performance of heterogeneous catalysts in thermochemical reactions depends on multiple factors, including surface area, accessibility of active sites, thermal and mechanical stability, resistance to deactivation, support acidity or basicity, compatibility with the reaction medium, and the inherent catalytic activity and selectivity.^38,39^ Marinič et al.^22^ developed a microkinetic model for the one-step HTL and upgrading of microalgae using NiMo/Al_2_O_3_, and observed partial deactivation due to catalyst surface coverage by unconverted biomass. Although postreaction catalyst evaluation was not performed in the present study, such deactivation could have affected catalytic efficiency. Future studies should incorporate this type of analysis to better understand catalyst performance. Another factor highlighted by Marinič et al.^22^ was the stirring speed. Their model indicated that 1000 rpm was sufficient to suppress external mass transfer limitations, significantly higher than the stirring rate used in this study (∼150 rpm), which may have contributed to the lower catalytic effect compared to results reported by other authors, such as.^12,40–43^ Finally, the compatibility of the catalyst with the reaction medium is also critical for conversion efficiency and selectivity. In some cases, aqueous media may result in lower conversions compared to organic solvents such as alcohols, mainly due to differences in polarity and hydrogen-donor capability.^38^ However, in HTL systems, the use of subcritical water provides unique advantages, acting simultaneously as a solvent, reactant, and catalyst medium, facilitating hydrolysis, decarboxylation, and deoxygenation reactions without requiring organic cosolvents. These characteristics make aqueous-based HTL particularly suitable for wet biomasses such as wastewater-grown microalgae.

### 3.3 Bio-oil Atomic Ratio H/C vs O/C

Figure 3 presents the Van Krevelen diagram showing the atomic H/C vs O/C ratio of the biomass, the bio-oil samples, and some fossil fuels. To complement the analysis of heteroatom evolution, an additional Van Krevelen plot (H/C vs N/C) was added to the Supporting Information (Figure S4).

**Figure 3:**
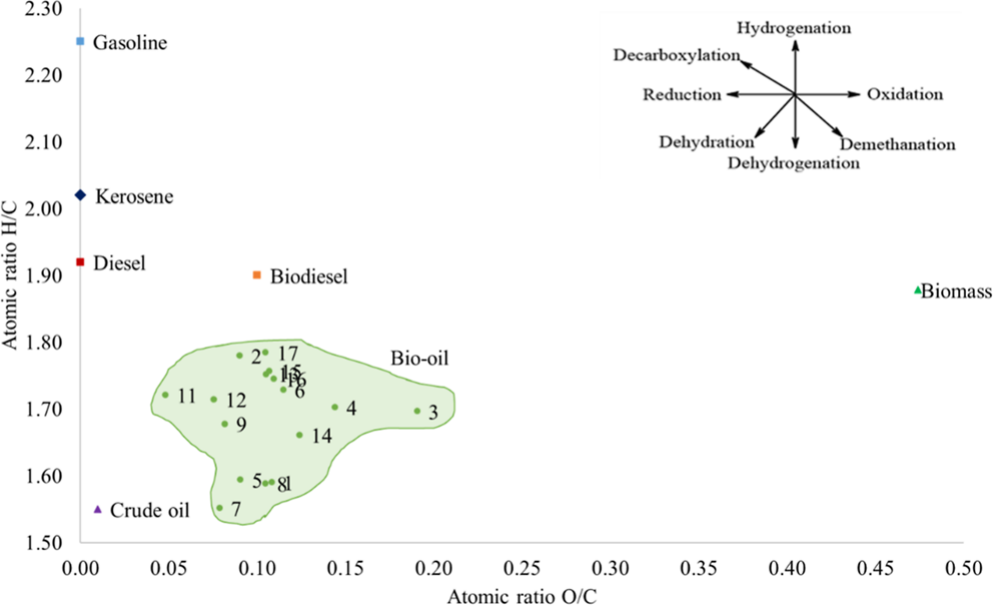
Van Krevelen diagram of the bio-oil produced in each batch.

The atomic H/C ratio of the bio-oil samples ranged from 1.49 to 1.78, slightly lower than that of the biomass (1.88). Similarly, the O/C ratio (0.05–0.19) was markedly reduced compared to the biomass (0.47). These changes are consistent with previous findings^8,26,30^ and indicate the occurrence of dehydration and deoxygenation reactions.^27,44^ During hydrothermal liquefaction and catalytic upgrading, dehydration typically proceeds via removal of hydroxyl groups from oxygenated intermediates, leading to the formation of unsaturated compounds and contributing to increased carbon content. Deoxygenation reactions, mainly decarboxylation and decarbonylation, further reduce oxygen levels while enhancing hydrocarbon fractions and heating value. Similar pathways have been reported for NiMo/Al_2_O_3_-catalyzed upgrading of algae bio-oil, promoting the conversion of fatty acids, esters, and oxygenated aromatics into more stable hydrocarbon molecules.^13,22,45–47^

A lower O/C ratio indicates reduced oxygen content and thus higher energy density, while a higher H/C ratio is generally associated with increased hydrogen saturation and higher heating value.^48^ The H/C ratio approached that of crude petroleum (1.5–2), while the O/C ratio still exceeded the ideal level (<0.02), especially in batch 11, which showed the best elemental profile. Despite the remaining oxygen and nitrogen contents, the similarity of H/C and O/C ratios to those of fossil fuels supports the potential of HTL as a biotechnology to convert biomass into an energy-dense material comparable to crude oil.^28,32^ Furthermore, the reduced H/C ratio may also indicate the formation of compounds with higher aromaticity.^49^ This trend suggests that secondary condensation, cyclization, and polymerization reactions occurred during hydrothermal processing and catalytic upgrading, leading to increased aromatic hydrocarbon content and decreased hydrogen saturation. Similar relationships between H/C ratio and aromaticity have been widely reported for NiMo/Al_2_O_3_-catalyzed upgrading of algae and lignocellulosic bio-oil.^13,15^ GC–MS analysis of the bio-oil (Figures S4 and S5, Supporting Information) confirmed the presence of aromatic hydrocarbons and substituted phenolics.

### 3.4 Bio-oil FTIR Analysis

The FTIR spectra shown in Figure 4 revealed similar patterns across all analyzed samples, but with variations in the relative intensities of the bands. This indicated that the samples contained similar functional groups.

**Figure 4:**
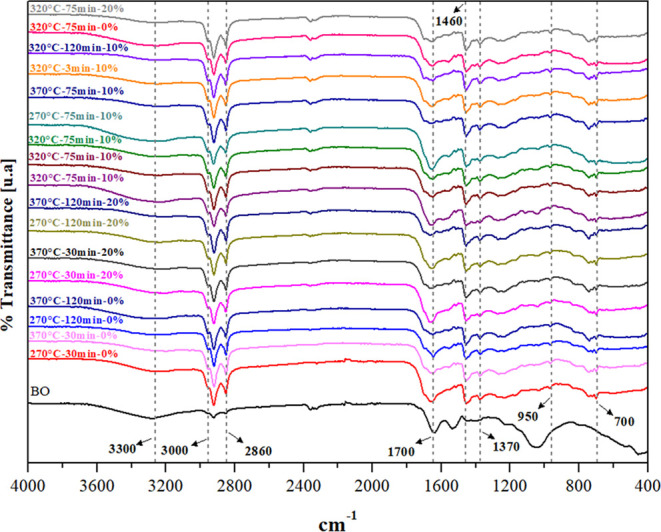
FTIR analysis of microalgae biomass (identified as BO) and bio-oil samples produced in each reaction.

The spectra revealed the presence of molecules such as phenols, ketones, esters, fatty acids, and nitrogen-containing compounds, formed during the thermal/catalytic conversion of microalgae into bio-oil. The broad band near 3300 cm^–1^ wavelength corresponded to O–H stretching from hydroxyl or carboxyl groups and N–H stretching in amino groups (traces of water, phenols, fatty acid amides, and N-containing heterocyclic compounds).^50,51^

The carbon and hydrogen content identified in the elemental analysis of the bio-oil (CHNS) (Table 3) was also confirmed in the FTIR analysis by the band presence between 2854 and 2965 cm^–1^, attributed to C–H stretching vibrations. In addition, bending vibrations of −CH_2_ at 1465 cm^–1^ and −CH_3_ at 1375 cm^–1^ confirmed the presence of aliphatic hydrocarbons (alkanes, alkenes, and alkynes without aromatic rings in their structure), which were also identified in the GC–MS results (see Section 3.5).^30,52^

Bands near 1700 cm^–1^ were associated with carbonyl (C=O) stretching vibration groups present in the bio-oil, indicating the presence of carboxylic acids, ketones, aldehydes, and esters.^51,53^ The esters in the bio-oil were confirmed by characteristic C–H and C–O stretching bands at wavelengths of 1457, 1263, and 1273 cm^–1^.^53^ The absence of bands in the 2000–2500 cm^–1^ range indicated the absence of compounds containing cumulative double or triple bonds.^50^ Additional bands at 650–900 cm^–1^ region were attributed to bending vibrations in the C–H bond of aromatic compounds.^54^

A comparison between bio-oil and microalgae biomass (BO) FTIR spectra showed a reduction in the intensity of bands near 3230 cm^–1^ (−OH), 1560 cm^–1^ (associated with aromatic C=C and carbonyl C=O stretching), 1273 cm^–1^ (ether groups), and 1045 cm^–1^ (C–O) after the HTL process. In contrast, the bands at 2922–2852 cm^–1^ (−CH_2_, –CH_3_) and 1458 cm^–1^ (C–H) increased in intensity. These changes can be explained by the reduction of carbonyl groups (e.g., –COOH) and oxygenated aromatic compounds originally present in the microalgae biomass during HTL, resulting in an increase in aliphatic structures such as alkanes and alkenes.^55^

### 3.5 Bio-oil GC–MS Analysis

Figure 5 summarizes the most abundant compounds identified in the bio-oil produced by HTL of wastewater-grown microalgae. Approximately 82 compounds were detected in each sample by GC–MS analysis. From these, the 20 compounds associated with the largest chromatographic peaks and similarity higher than 70% relative to the reference database were selected. These compounds accounted for approximately 72% of the total chromatographic peak area. The identified compounds were grouped according to molecular structure and functional groups. Complete chromatograms and detailed compound lists for reactions 7 and 11, corresponding to the highest bio-oil yield and highest HHV, respectively, are provided in the Supporting Information (Figures S5 and S6 and Tables S6 and S7).

**Figure 5:**
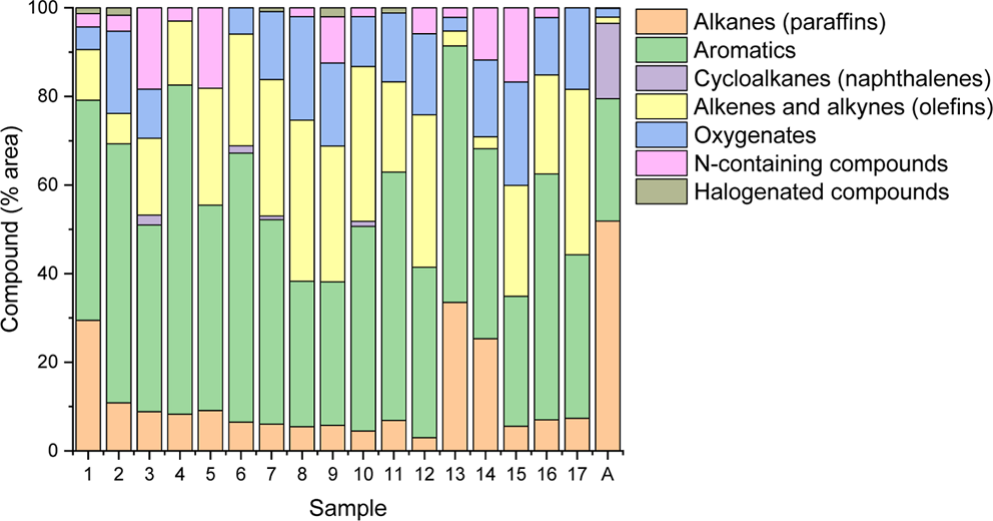
Major compounds identified by GC–MS in the bio-oil produced from wastewater-grown microalgae via HTL conducted at 270–370 °C and approximately 5–21 MPa, using NiMo/Al_2_O_3_ catalyst (20 most abundant peaks, similarity ≥70%). Compounds are classified by molecular structure and functional groups. Note: Column “A” represents the composition of aviation kerosene derived from petroleum, adapted from Jeon et al. (2024).

Considering the compounds corresponding to the 20 most abundant peaks, on average, 80.90% were hydrocarbons. Among these, aromatics represented 47.42%, followed by alkenes and alkynes (olefins) at 22.34%, and alkanes (paraffins) accounting for 10.80% of the compounds on average. Oxygenated compounds made up 12.25% on average, and nitrogen-containing compounds accounted for 5.52%.

The predominance of hydrocarbons (80.90%), particularly aromatics (47.42%), confirmed the observation in Section 3.4—Figure 4, regarding the reduction in the H/C atomic ratio, which is associated with the formation of aromatic compounds.^49^ The oxygenated (12.25%) and nitrogenated (5.52%) compound contents indicate the need for further refining to reduce these undesirable components. The bio-oil exhibited desirable fractions for biokerosene, such as alkanes, cycloalkanes, and aromatics; however, controlling the proportion of aromatics and naphthenes is essential to avoid carbonaceous deposits in aircraft turbines. The presence of oxygenated and nitrogenated compounds must be minimized to improve the thermal and chemical stability of the fuel. Thus, upgrading the bio-oil and improving its physicochemical characteristics remains a challenge to be overcome in the context of biofuels.^17^

To address this challenge, in addition to one-step HTL and upgrading (as evaluated in this study), other strategies have also been researched, such as co-HTL, the use of alternative solvents to water as the reaction medium, and two-stage HTL.^14^

Comparing the bio-oil obtained via one-step HTL and upgrading with NiMo/Al_2_O_3_ and petroleum-derived jet fuel, further refining is necessary to increase the content of alkanes and cycloalkanes, and to reduce the presence of aromatic hydrocarbons, alkynes, alkenes, and compounds containing oxygen, nitrogen, and halogens.^56^ This additional refining can be carried out later and may involve mild conditions to remove reactive compounds such as olefins and some sulfur and nitrogen compounds, or more severe conditions to saturate aromatic rings and remove almost all sulfur and nitrogen compounds.^57^ One possibility is coprocessing the bio-oil with petroleum intermediate distillates. This is the most widely accepted method for blending renewable and fossil fuels and can be performed in conventional refineries, resulting in a drop-in fuel (ready-to-use) with a low carbon footprint and economic feasibility.^2^ Thus, it is important to continue developing research focused on improving bio-oil quality, whether through catalyst use or other approaches as mentioned above. For HTL bio-oil from microalgae to become commercially competitive, its yield must be increased, and its quality enhanced.

### 3.6 HTL Byproducts Analysis

#### 3.6.1 Aqueous Phase

The water-soluble compounds (aqueous phase) accounted for 11.75 to 30.22% of the products obtained in the reactions performed. Figure 6 presents the organic compounds present in the aqueous phase generated in each of the HTL reactions.

**Figure 6:**
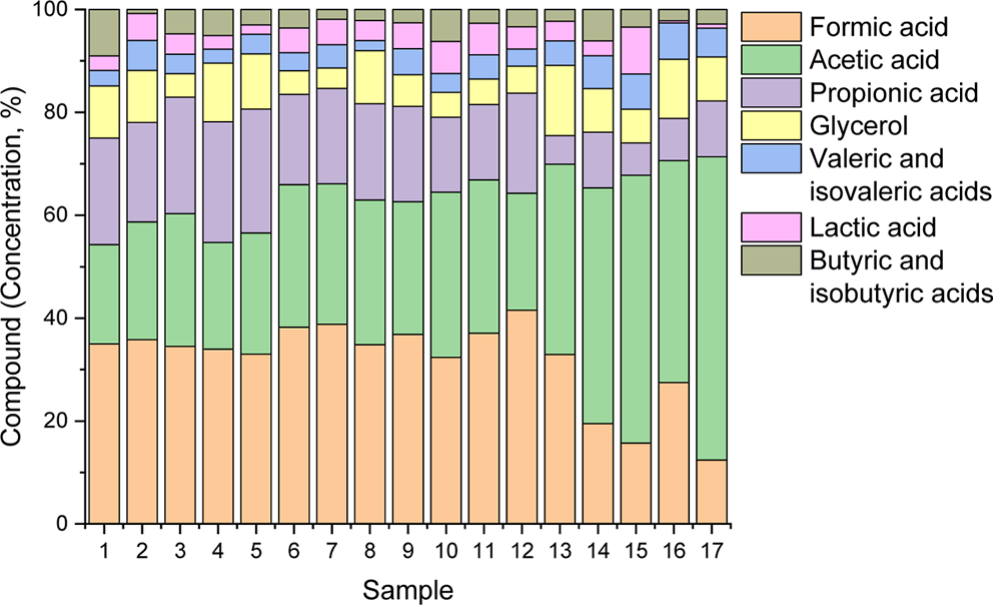
Compounds detected in the aqueous phase.

The predominant compounds in the aqueous phase, in all treatments, were formic acid (26.414 mg/L on average), acetic acid (24.076 mg/L on average), and propionic acid (13.743 mg/L on average), representing approximately 33%, 30%, and 17% of the relative composition of the detected compounds, respectively. Increasing temperature had a significant effect (*p*-value < 0.01) on increasing acetic acid formation and reducing formic acid content. Thermal decomposition of formic acid occurs at temperatures above 320 °C.^58^

Short-chain organic acids, such as those found (formic, acetic, and propionic acids), are commonly reported in the aqueous phase of microalgae HTL^59^ and other biomass feedstocks.^13,60^ These acids are intermediate products formed from carbohydrate degradation, also known as decarboxylation.^61^ They can be extracted and used in the polymer, leather, and dyeing industries.^13,41^

Glycerol, also identified in the aqueous phase (6.417 mg/L on average; 8% of the detected compounds), stands out for its potential to be used as a substrate for the production of biohydrogen (bio-H_2_) and biomethane (bio-CH_4_).^62^ Additionally, the aqueous phase can serve as a source of nutrients for microalgae growth. However, strategies such as dilution and mixing with other media need to be applied to mitigate its negative effects.^51,63,64^

#### 3.6.2 Solid Phase

The solid residues ranged from 19.09 to 28.52% of the reaction yield. The C content in the solids generated ranged from 11.73% to 27.07%, H content ranged from 1.76% to 4.51%, and N content from 1.34% to 2.17% (Table S8, in the Supporting Information). The composition of solids generated from algae HTL varies from 3 to 54% C, 1 to 7% H, 1 to 11% N, 2 to 22% O.^65^ The HHV can range from 2 to 25 MJ kg^–1^. C and H contents of 47.8% and 6.1%, respectively, were found by Mishra and Mohanty^30^ in the co-HTL of sludge and microalgae biomass, both derived from wastewater treatment. The solids produced had an estimated HHV of 17.23 MJ kg^–1^, indicating potential use as a feedstock for bioenergy production. Another possible use is the extraction of interesting compounds, such as the acid extraction for P recovery, followed by combustion of the residual solid.^31^ Also, the use of the solid phase as an adsorbent to immobilize metals from contaminated wastewater.^66^

#### 3.6.3 Gaseous Phase

The gas yield varied from 8.72 to 15.72% of the products obtained in the reactions performed. CO_2_ was the predominant gas in all HTL reactions, accounting for 100% of the gaseous product. Zhou et al.^65^ also reported that more than 90% of the gas generated during algae HTL consisted of CO_2_, suggesting decarboxylation reactions.

In the reactions carried out, between 0.5 and 0.9 dm^3^ of CO_2_ was generated. This gas can be recirculated in microalgae cultivation^13,47,67^ or used in technologies that apply supercritical CO_2_, such as in the extraction of bioactive compounds or in separation and extrusion processes. Additionally, it can be used as a heat transfer fluid in power cycles or in carbon capture and storage systems.^13^ The gas generated during HTL is also a source of biogenic CO_2_ (originating from organic sources) and may contribute to the reduction of CO_2_ emissions, in line with global decarbonization goals.^68,69^

### 3.7 Limitations and Future Perspectives

The results obtained in this study demonstrate the potential of one-step HTL and catalytic upgrading for converting wastewater-grown microalgae into bio-oil with characteristics similar to fossil fuels. However, some limitations should be acknowledged. The experimental design was focused on identifying the influence of temperature, reaction time, and catalyst concentration on bio-oil yield and composition, rather than evaluating catalyst stability or long-term performance. Postreaction catalyst characterization, which would provide insights into deactivation mechanisms such as surface fouling or metal leaching, was not performed. Additionally, a full elemental mass balance could not be established because CHNSO analysis of the aqueous phase was not performed, and this limitation should be addressed in future studies.

Although this study focused on process optimization, catalyst stability and possible deactivation during hydrothermal liquefaction and upgrading were not evaluated. Future work should include postreaction characterization of the spent catalyst (e.g., XRD, BET, SEM–EDS, TGA) to investigate structural changes, surface area loss, or coke deposition. These analyses are essential for assessing the catalyst’s reusability and the long-term sustainability of the process.

Previous studies (e.g., Zhou et al., Mukundan et al., Magalhães et al., Marinič et al., and Borazjani et al.^13,22,45–47^ ) have reported that NiMo/Al_2_O_3_ catalysts in hydrothermal environments can undergo partial deactivation due to organic deposition, inorganic accumulation from the biomass, or structural modifications under reaction conditions. Considering that wastewater-grown microalgae have higher ash content and more complex inorganic composition compared to pure-culture biomass, catalyst interaction effects may differ and warrant dedicated investigation.

Future work should therefore focus on: (i) detailed postreaction catalyst characterization, (ii) assessment of catalyst regeneration and reuse, (iii) evaluation of process performance in continuous-flow reactors, and (iv) integration of catalyst and bio-oil upgrading strategies to reduce heteroatom content and improve fuel stability.

Additionally, techno-economic assessment (TEA) and life cycle assessment (LCA) will be essential to determine the economic feasibility and environmental performance of this route at larger scales. These studies will help to identify key cost drivers, emissions reductions potential, and process bottlenecks, supporting future decisions regarding process scale-up and industrial implementation.

## 4 Conclusions

The highest bio-oil yield achieved was 23.07% at 320 °C for 30 min using 10% NiMo/Al_2_O_3_ catalyst, while the highest higher heating value (41.77 MJ kg^–1^) was obtained under similar conditions with a longer reaction time. Temperature was the main factor influencing bio-oil yield, and the catalyst played a crucial role in sulfur reduction. The optimal set of operational parameters, around 324 °C with 15% NiMo/Al_2_O_3_ catalyst, provided a favorable balance between high bio-oil yield and low sulfur content. Considering energy efficiency, the shortest tested reaction time (30 min) was recommended, as longer durations did not significantly improve the product.

The resulting bio-oil exhibited a chemical composition similar to crude petroleum, with high energy potential, although further refining was still required to meet SAF specifications, particularly to increase the alkane fraction and reduce the presence of aromatics and oxygenated compounds.

Characterization of the aqueous, solid, and gaseous phases also revealed promising routes for the valorization of byproducts, expanding the potential for full biomass utilization.

Therefore, this research reaffirmed HTL as a viable and strategic technological route for converting algae biomass into advanced biofuel. The results represent a significant step toward more efficient and sustainable processes aligned with future clean energy demands. Further research should focus on catalyst optimization, impurity reduction, and process scalability, bringing this technology closer to industrial application. In addition, comprehensive technical, economic, and environmental feasibility studies are essential to assess the full potential of SAF precursor production from microalgae.

## Supplementary Material



## Notes

Wastewater characterization; biomass characterization methodology; experimental design; catalyst preparation and characterization; bio-oil elemental composition; statistical analysis; Van Krevelen diagram; chromatograms and the compounds detected in batches 7 and 11; solid phase characterization (PDF)
