# Supplementary material for: One-step Hydrothermal Liquefaction and Catalytic Upgrading of Wastewater-Grown Microalgae for Potential Sustainable Aviation Fuel Precursors
Source: ACS Omega. 2026 Jan 20;11(4):6073–83. doi: 10.1021/acsomega.5c10732 (PMC12878768; doi:10.1021/acsomega.5c10732)
Supplement: Supplementary file 1 [file ao5c10732_si_001.pdf]

One-step hydrothermal liquefaction and catalytic upgrading of wastewater-grown microalgae for potential sustainable aviation fuel precursors

Bianca Barros Marangon [a,], Jackeline de Siqueira Castro [a], Fabiane Carvalho Ballotin [b], Laís Santos Silva [c], Paula Assemany [d], Eduardo Aguiar do Couto [d], Thiago Abrantes Silva [a], Maurino Magno Jesus Junior [c], Vinícius José Ribeiro [e], José Ivo Ribeiro Júnior [f], Ana Márcia Carvalho [g], Sarah de Paiva Silva Pereira Pinheiro [c], Sergio Antonio A Fernandes [c], Maria Lúcia Calijuri [a].*

** Corresponding author*

[a] Department of Civil Engineering, Federal University of Viçosa (Universidade Federal de Viçosa), Viçosa, MG, Brazil.

[b] Department of Chemistry, Federal University of Lavras (Universidade Federal de Lavras), Lavras, MG, Brazil.

[c] Department of Chemistry, Federal University of Viçosa (Universidade Federal de Viçosa), Viçosa, MG, Brazil.

[d] Department of Environmental Engineering, Federal University of Lavras (Universidade Federal de Lavras), Lavras, MG, Brazil.

[e] Department of Soils, Federal University of Viçosa (Universidade Federal de Viçosa), Viçosa, MG, Brazil.

[f] Department of Statistics, Federal University of Viçosa (Universidade Federal de Viçosa), Viçosa, MG, Brazil.

[g] Department of Forestry Engineering, Federal University of Viçosa (Universidade Federal de Viçosa), Viçosa, MG, Brazil.

Supplementary material

Wastewater characterization

The wastewater from the meat-processing industry presented the characteristics shown in Table S1.

Table S1. Characterization of the wastewater from the meat-processing industry

Parameter	Symbol	Value ($mg \cdot L^{-1}$)	Method/Reference
Total suspended solids	TSS	545.1	APHA et al. (2023)
Volatile suspended solids	VSS	471.5	APHA et al. (2023)
Ammoniacal nitrogen	$N–NH_3$	15.68	APHA et al. (2023)
Soluble phosphorus	Ps	0.31	APHA et al. (2023)
Soluble chemical oxygen demand	CODs	58.60	APHA et al. (2023)
Total organic carbon	TOC	258.5	Shimadzu TOC 5000A analyzer

Biomass characterization methodology

The analytical methods used for biomass characterization are summarized in Table S2.

Table S2. Analytical methods for biomass characterization

Parameter / Analysis	Method / Equipment	Reference
Phytoplankton identification	Inverted microscopy	Komarek e Fott (1983), Parra et al. (1982), APHA (2012), Utermöhl (1958), and Wetzel and Likens (1991).
Lipid content	Soxhlet extraction with hexane (neutral lipids, 5 h 30 min) and ethanol (membrane lipids, 2 h 30 min) after cell disruption using a Tecnal TE-099 mill and fat extractor (Tecnal TE-044-8/50)	AOAC (2000)
Protein content	Kjeldahl (NKT) method with nitrogen-to-protein conversion factor of 6.25	APHA et al. (2023)
Carbohydrate content	Determined by difference: 100% – (lipids + proteins + ash + moisture)	Wang et al. (2018)
Proximate analysis (moisture, ash, volatile matter, fixed carbon)	ASTM D3172; sieving at 0.425 mm and 0.250 mm (TYLER MESH 35–60)	ASTM (2021)
Elemental composition (C, H, N, S)	Elemental analyzer (Elementar Vario Micro Cube); O calculated by difference	—
Higher heating value (HHV)	Estimated from elemental composition using: HHV ($MJ \cdot kg^{-1}$) = 33.86 × C + 144.4 × (H – O/8) + 9.428 × S	Perry and Chilton (1973)
Atomic ratios (H/C, O/C)	Calculated from CHNS composition adjusted for atomic mass ratios (H:1/12; O:16/12; N:14/12; S:32/12)	—

Experimental design

Reactions performed, based on the experimental planning *Central Composite Design*, *Face Centered*, with triplicate at the central point and 6 axial points (Table S3).

Table S3. Experimental design.

Factor	Level		
	-1	0	+1
Temperature (°C)	270	320	370
Time (min)	30	75	120
Catalyst (% w.w^{-1})	0	10	20

Catalyst preparation and characterization

The nickel–molybdenum catalyst supported on alumina (NiMo/Al_2O_3) was prepared by the two-step wet impregnation method using aqueous solutions of nickel nitrate hexahydrate ($Ni(NO_3)_2 \cdot 6H_2O$, 98% purity) and ammonium heptamolybdate tetrahydrate ($(NH_4)_6Mo_7O_{24} \cdot 4H_2O$, 81–83% purity), as described by Jafarian et al. (2019) and Moazezi et al. (2022) to obtain a catalyst with 2 wt% of Ni and 12 wt% of Mo. The catalyst was then dried in an oven at 120 °C for 5 hours and calcined in a muffle furnace at 550 °C for 3 hours.

Catalyst surface images were obtained using scanning electron microscopy (SEM) (JSM-6010LA) with an accelerating voltage of 15.0 kV. The sample was coated with gold prior to imaging. Energy-dispersive X-ray spectroscopy (EDS) was performed alongside SEM to identify the main elements present. The catalyst particles ranged from 2 to 5 µm, composed of small agglomerates supported on Al_2O_3, which exhibited hexagonal planes (Figure S1), mainly attributed to the presence of Mo. The elements Ni and Mo were found to be dispersed across the surface of the support material (Al_2O_3) (Figure S2). The Ni content was measured at 2.1%, consistent with the target value (2 wt%), and the Mo content was 15.3%, also close to the expected value (12 wt%).

The thermal degradation profile of the material was obtained through thermogravimetric analysis (TGA) (DTG-60H, Shimadzu) under a nitrogen atmosphere with a constant flow rate of 50 mL·min^{-1}, across a temperature range from 100 to 800 °C. The catalyst demonstrated thermal stability up to 800 °C, with a total weight loss of approximately 4% (Figure S3). Three stages of mass loss were observed: the first two (below 200 °C and between 200 and 400 °C) were attributed to the release of weakly and strongly bound

water, respectively; the third stage, from 500 to 800 °C, was likely associated with hydroxyl groups loss (Asiedu et al., 2020), which may continue beyond this temperature (Al-Iessa et al., 2023).

Images of the catalyst surface obtained by scanning electron microscopy (SEM) (Figure S.1). Energy dispersive spectroscopy (EDS) to identify the main elements present in the catalyst (Figure S.2).

Catalyst degradation curve and mass variation rate (thermogravimetric derivative, DTG), obtained by thermogravimetric analysis (TGA) (Figure S.3).

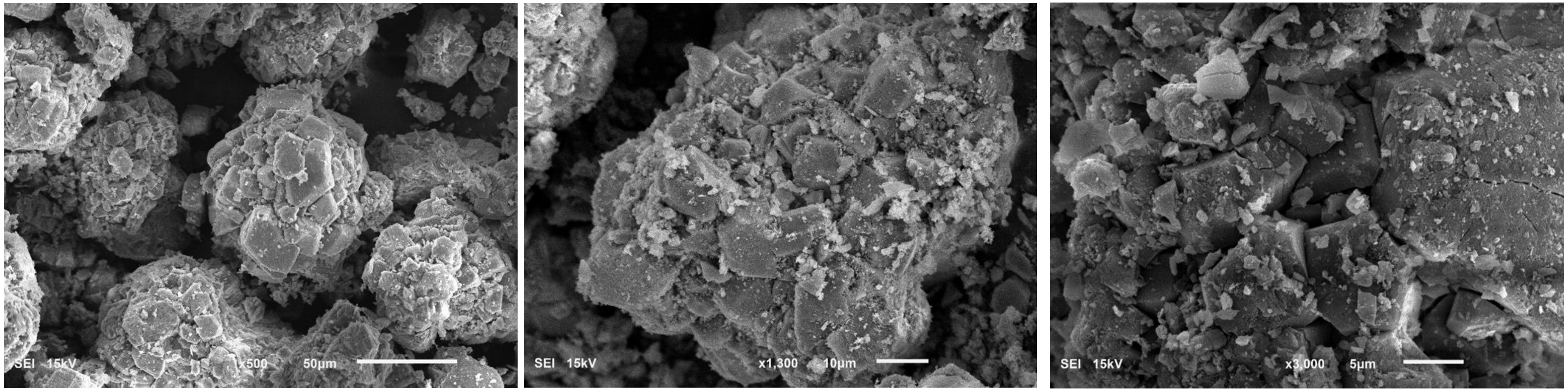

Figure S1. SEM images of NiMo/Al_2O_3 catalyst surface, at three magnifications x500, x1,300 and x3,000.

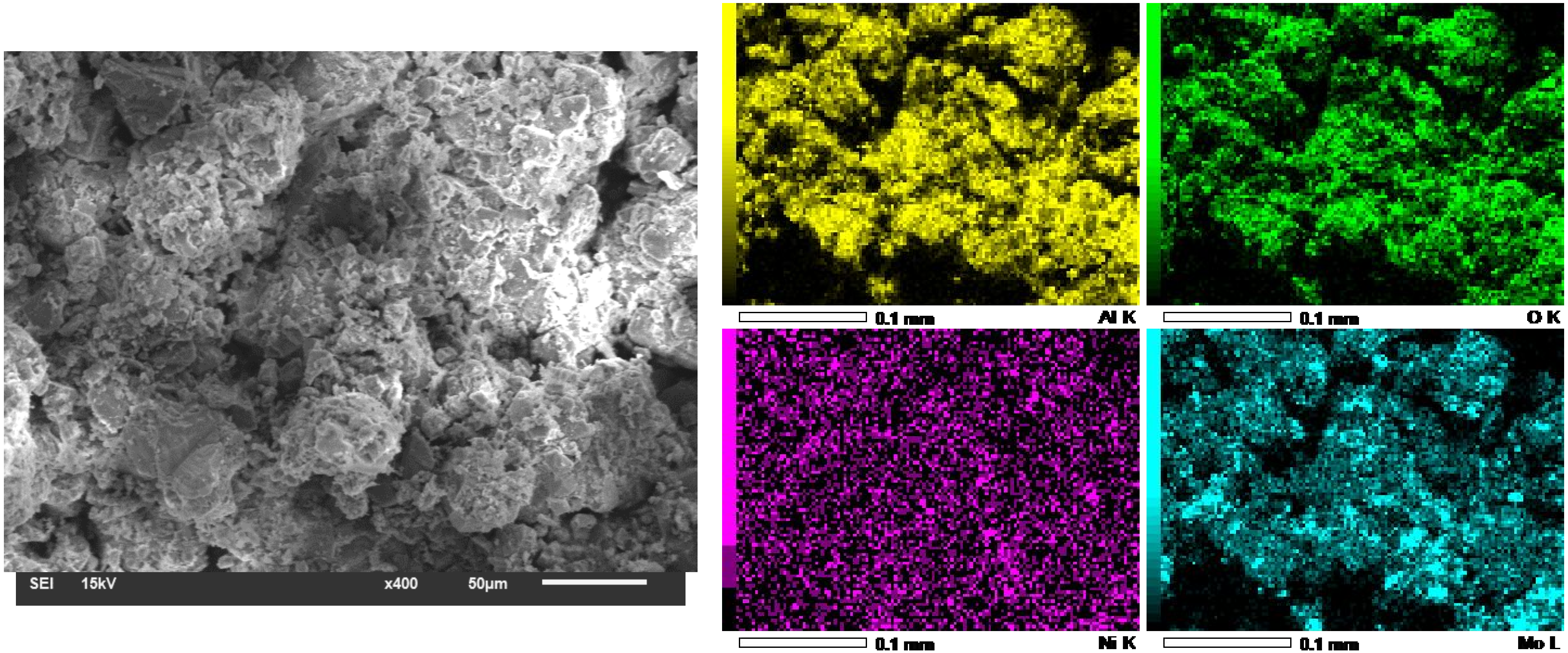

Figure S2. SEM images of NiMo/Al_2O_3 catalyst surface, with identification of the main elements by EDS.

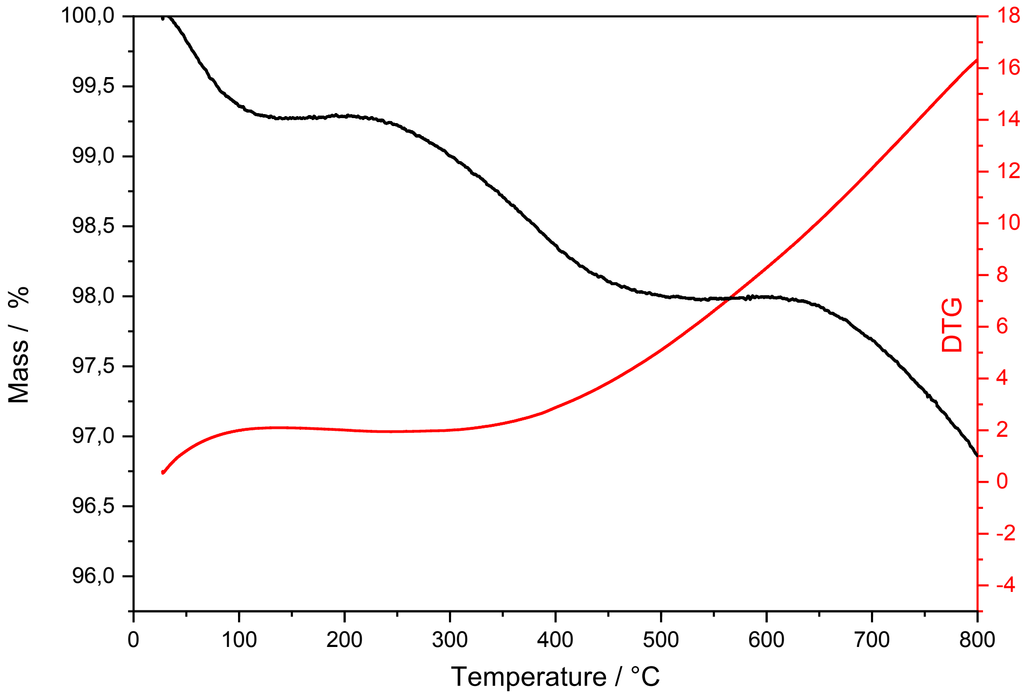

Figure S3. TG/DTG curves of $NiMo/Al_2O_3$ catalyst.

Bio-oil elemental composition

Table S4 presents the carbon (C), hydrogen (H), nitrogen (N), sulfur (S), and oxygen (O) contents of the bio-oils produced in each experimental run. Elemental analysis was performed using an Elementar Vario Micro Cube analyzer, and oxygen was calculated by difference.

Table S4. Elemental composition (wt%, dry basis) of bio-oils obtained under different reaction conditions.

Batch ID	Experimental condition			Elemental analysis (wt%)				
	Temperature (°C)	Time (min)	Catalyst (wt%)	C	H	N	S	O
1	270	30	0	72.90	9.66	5.50	1.41	10.53
2	270	120	0	73.50	10.90	5.26	1.50	8.84
3	270	75	10	66.50	9.40	6.52	0.67	16.91
4	270	30	20	70.00	9.93	5.89	0.72	13.46
5	270	120	20	74.60	9.91	5.89	0.58	9.02
6	320	75	0	72.20	10.40	5.46	0.89	11.05
7	320	30	10	76.10	9.84	5.28	0.79	7.99
8	320	75	10	73.60	9.74	5.61	0.76	10.29
9	320	75	10	75.10	10.50	5.55	0.66	8.19
10	320	75	10	75.50	9.38	5.84	0.65	8.63
11	320	120	10	78.10	11.20	5.06	0.62	5.02
12	320	75	20	75.60	10.80	5.38	0.62	7.60
13	370	120	0	72.60	10.60	5.00	1.61	10.19
14	370	30	0	71.30	9.87	5.74	1.31	11.78
15	370	75	10	73.10	10.70	5.01	0.79	10.40
16	370	30	20	72.90	10.60	5.18	0.69	10.63
17	370	120	20	73.30	10.90	4.85	0.71	10.24

Statistical analysis

Table S5 presents the regression coefficients estimated by the adjusted statistical model for the response variables tested (bio-oil yield and C, H, N, S, and O contents) as a function of the evaluated operational parameters (temperature, reaction time, and catalyst).

Table S5. Estimated coefficients of the adjusted statistical model.

Response variable	Regression equation	R^2
Bio-oil yield (%)	$-166{,}4 + 1{,}128^{*}t + 0{,}766c - 0{,}001685^{*}t^2 - 0{,}002400^{*}tc$	84.46%
C content (%)	73.35 (mean value)	–
H content (%)	10.25 (mean value)	–
N content (%)	5.47 (mean value)	–
S content (%)	$8.48 - 0.0460t - 0.0749^{*}c + 0.000073^{*}t^2 + 0002043^{*}c^2$	86.70%
O content (%)	10.04 (mean value)	–

* = Significant by Student's t-test at 5% of significance; t = temperature (270 °C ≤ t ≤ 370 °C) and c = catalyst (0% ≤ c ≤ 20%).

Van Krevelen diagram

Figure S4 presents the Van Krevelen diagram showing the atomic H/C vs. N/C ratio of the biomass and the bio-oil samples.

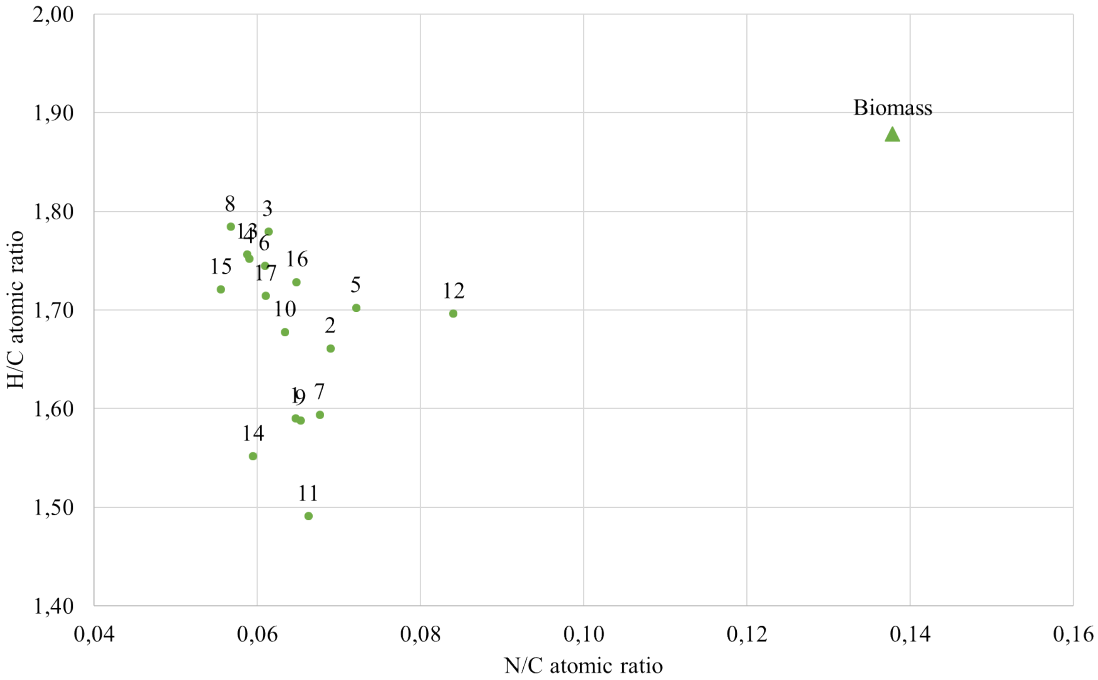

Figure S4. Van Krevelen diagram of the bio-oil produced in each batch.

Chromatograms and the compounds detected in batches 7 and 11

The chromatograms and the compounds detected in reactions 7 and 11, which yielded the highest bio-oil production and the highest HHV, respectively, are presented in Figures S5 and S6 and Tables S6 and S7.

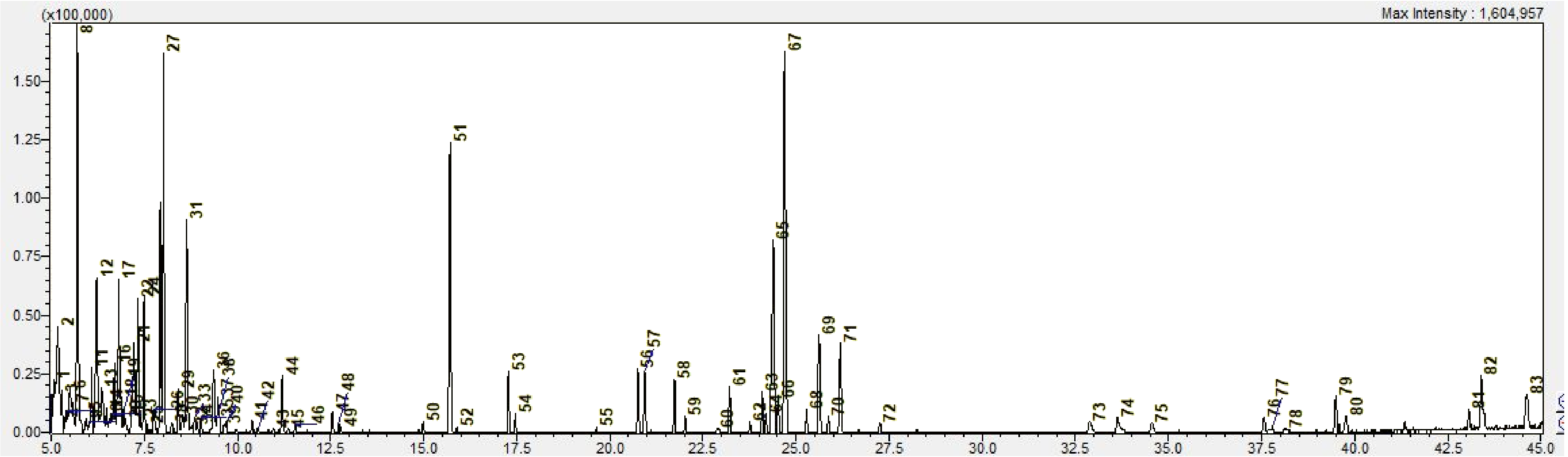

Figure S5. Chromatogram of batch 7

Table S6. List of compounds detected in batch 7.

Peak number	Retention time	Area	Area (%)	Compound name	Similarity (%)
1	5.070	41051	0.69	1-chlorooctane	76
2	5.177	208222	3.51	Cumene	83
3	5.300	38580	0.65	8-Benzoyloctanoic acid	81

4	5.365	7835	0.13	2,4,4-trimethylpent-2-ene	81
5	5.423	6718	0.11	4,4’-dimethyl-1,1’-bi(cyclohexane)	80
6	5.511	51155	0.86	2-(3-hydroxy-2-nitrocyclohexyl)-1-phenylethan-1-one	72
7	5.575	23933	0.40	5-bromo-2,3-dimethylpent-2-ene	77
8	5.705	496827	8.39	1,2,4-trimethylbenzene	74
9	5.933	14658	0.25	furan-2(5H)-one	81
10	6.007	3367	0.06	3-allylcyclopent-1-ene	75
11	6.091	53305	0.90	3,3,5-trimethylheptane	93
12	6.223	233330	3.94	Mesitylene	84
13	6.356	64602	1.09	Octylcyclohexane	89
14	6.487	15619	0.26	(Z)-prop-1-en-1-ylbenzene	93
15	6.618	4025	0.07	3,3-dimethylbutan-1-ol	88
16	6.701	53742	0.91	4-methylpentyl 2-phenylpropanoate	80
17	6.817	199281	3.36	Tert-butyl 2-phenylacetate	79
18	6.875	20929	0.35	(1r,4r)-1-methyl-4-(prop-1-en-2-yl)cyclohexane	81
19	6.952	15848	0.27	2-Bromononane	86
20	6.995	2611	0.04	1,2-dihydro-3H-indazol-3-one	90
21	7.222	133540	2.25	p-Cymene	76
22	7.335	131772	2.22	1-methyl-4-(prop-1-en-2-yl)cyclohexa-1,3-diene	86
23	7.399	3449	0.06	N-((trifluoromethyl)thio)formamide	77
24	7.490	132883	2.24	4,7-dimethylundecane	92
25	7.585	3855	0.07	octan-4-yl 2,2,2-trifluoroacetate	86
26	7.754	28728	0.48	1-(p-tolyl)pentan-1-one	82
27	8.015	645440	10.89	1,2,4,5-tetramethylbenzene	95
28	8.246	9296	0.16	Methyl (3-methylbut-2-enoyl)glycinate	89
29	8.405	54772	0.92	(2-methylcyclopropyl)benzene	85
30	8.527	23998	0.41	tert-butyl (4-vinylphenyl) carbonoperoxoate	73
31	8.643	284668	4.80	1,2,4,5-tetramethylbenzene	88
32	8.777	6761	0.11	2,5-dimethylbenzyl 2,4-dimethylbenzoate	91

33	8.848	14800	0.25	(2,3-dimethylbutane-2,3-diyl)dibenzene	77
34	8.921	3740	0.06	N-methylaniline	97
35	9.027	13132	0.22	3-(4-methylbenzoyl)-2-thioxo-2,3-dihydrothiazol-4-yl 4-methylbenzoate	89
36	9.358	98067	1.66	Azulene	85
37	9.474	42486	0.72	1,1,2,2-tetramethoxyethene	84
38	9.525	13113	0.22	1,4-diethyl-2-methylbenzene	92
39	9.649	5337	0.09	N-(4-(dimethylamino)benzyl)thiazol-2-amine	76
40	9.708	7071	0.12	2-(6,6-dimethylbicyclo[3.1.1]hept-2-en-2-yl)ethyl (E)-2-methylbut-2-enoate	100
41	10.390	12897	0.22	4-propylpyridine	91
42	10.548	4781	0.08	(fluorocarbonyl)sulfurimidous difluoride	100
43	10.967	3900	0.07	5,6,7,8-tetrahydronaphthalen-2-ol	100
44	11.200	65746	1.11	6-methylheptan-1-ol	81
45	11.370	3320	0.06	Allyl butyl oxalate	100
46	11.561	8989	0.15	2-methylbenzaldehyde oxime	92
47	12.552	20002	0.34	(S)-3-methylpentan-1-ol	86
48	12.719	5205	0.09	2-methylbutyl formate	85
49	12.771	4195	0.07	N-methyl-3-propyl-1,2,4-thiadiazol-5-amine	95
50	14.988	10956	0.18	1,2,3-Trimethyldiaziridine	91
51	15.708	375001	6.33	n-Tridecan-1-ol	93
52	15.891	3888	0.07	but-3-en-2-ol	94
53	17.275	67753	1.14	6-methylheptan-1-ol	89
54	17.464	14142	0.24	4,5-dimethyloctane	85
55	19.641	3811	0.06	but-3-en-2-ol	95
56	20.757	80149	1.35	Undecyl 2,2,2-trichloroacetate	86
57	20.938	83587	1.41	3,3-dimethylhexane	91
58	21.737	60483	1.02	1-methyl-2-(3-methylpentyl)cyclopropane	86
59	22.025	12277	0.21	4-methylhexan-1-ol	89
60	22.903	6161	0.10	4-methyl-1,3-oxathiolane	100
61	23.217	54044	0.91	3,7-dimethyloctyl 2,2,2-trifluoroacetate	88

62	23.765	9851	0.17	1,2,3-Trimethyldiaziridine	91
63	24.094	47634	0.80	8-methyldec-1-ene	85
64	24.193	15152	0.26	2,3-dimethylhexane	83
65	24.390	368664	6.22	(7R,11R,E)-3,7,11,15-tetramethylhexadec-2-ene	93
66	24.516	32688	0.55	(R)-3,7-dimethylocta-1,6-diene	83
67	24.695	591048	9.98	(7R,11R,E)-3,7,11,15-tetramethylhexadec-2-ene	92
68	25.286	24220	0.41	Methyl 2-(hydrazinecarbonyl)-1H-imidazole-1-carboxylate	79
69	25.619	144959	2.45	3,7,11-trimethyldodeca-2,4-diene	82
70	25.870	19618	0.33	tridec-1-yn-4-ol	82
71	26.185	125815	2.12	3,7,11-trimethyldodeca-2,4-diene	78
72	27.252	13454	0.23	1,2-dineopentyl-1l6,2l4-disulfane-1,1,2-trione	100
73	32.886	29600	0.50	2-ethoxyethyl (2-methoxyethyl) carbonate	93
74	33.637	41508	0.70	Butyraldehyde O-methyl oxime	94
75	34.552	22612	0.38	(5R,6S)-decane-5,6-diol	100
76	37.561	25895	0.44	1-isothiocyanato-2-methylpropane	77
77	37.792	11705	0.20	3-methoxy-3-methylbutan-2-one	90
78	38.136	10406	0.18	Octane-4,5-diol	100
79	39.485	50380	0.85	dodecane-1,2-diol	81
80	39.771	37928	0.64	5-ethyl-3,5-dimethyloxazolidine-2,4-dione	77
81	43.074	19379	0.33	dodecanedinitrile	79
82	43.399	95135	1.61	nonadecan-1-ol	83
83	44.609	73509	1.24	Nonyl prop-1-en-2-yl carbonate	77

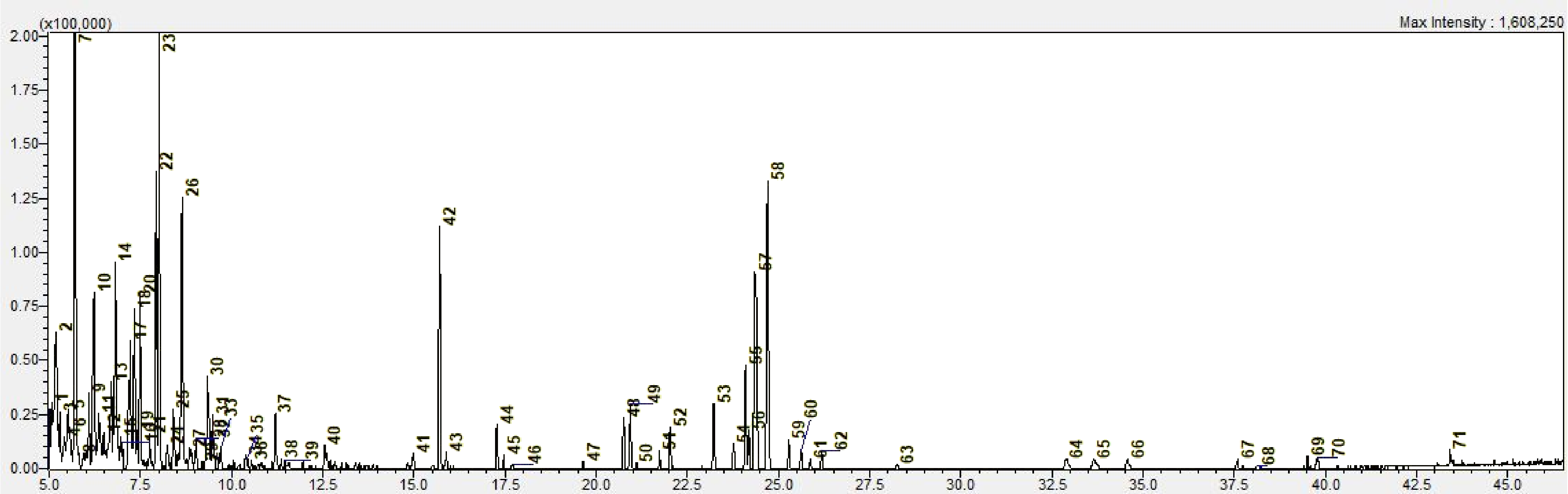

Figure S6. Chromatogram of batch 7

Table S7. List of compounds detected in batch 7.

Peak number	Retention time	Area	Area %	Compound name	Similarity (%)
1	5.075	62723	0.87	1-chlorooctane	71
2	5.183	267701	3.71	Cumene	85
3	5.291	45279	0.63	1,2,3-trimethylbenzene	82
4	5.426	12057	0.17	(1R,2R)-1-methyl-2-nonylcyclohexane	84
5	5.522	68865	0.95	(1S,3S)-1-isopropyl-3-methylcyclohexane	87
6	5.575	33217	0.46	Octanenitrile	82
7	5.706	689558	9.56	Decan-2-yl 2-phenylbutanoate	73

8	5.825	15765	0.22	2,2,2-trifluoroacetic anhydride	78
9	6.093	61296	0.85	3,3-dimethyloctane	92
10	6.226	296911	4.11	1,2,3-trimethylbenzene	87
11	6.360	113774	1.58	Decylcyclohexane	91
12	6.491	52904	0.73	Indane	89
13	6.700	176340	2.44	4-methylpentyl 2-phenylpropanoate	80
14	6.818	369996	5.13	Tert-butyl 2-phenylacetate	77
15	6.950	36062	0.50	1-iodononane	86
16	7.005	21639	0.30	Diphenethylamine	77
17	7.223	216763	3.00	Heptan-2-yl 2-phenylbutanoate	75
18	7.335	202366	2.80	2-ethyl-1,4-dimethylbenzene	88
19	7.410	30768	0.43	N'-(5-bromo-3-(tert-butyl)-2-hydroxybenzylidene)spiro[2.3]hexane-1-carbohydrazide	61
20	7.492	180886	2.51	Undecane	93
21	7.753	36445	0.51	1-(p-tolyl)pentan-1-one	88
22	8.015	869339	12.05	1,2,4,5-tetramethylbenzene	95
23	8.248	32939	0.46	(Z)-hex-3-en-1-yl (E)-2-methylbut-2-enoate	87
24	8.403	100785	1.40	(2-methylcyclopropyl)benzene	86
25	8.643	437015	6.06	1,2,3,4-tetramethyl-5-methylenecyclopenta-1,3-diene	91
26	8.850	70869	0.98	3-(methylthio)propane-1,2-diol	81
27	9.029	28793	0.40	2-isopropyl-5-methyl-4-(((4-methylbenzoyl)oxy)imino)cyclohexa-2,5-dien-1-one	77
28	9.200	5921	0.08	2-methyl-1,3,5,2,4,6-triazatriborinane	99
29	9.340	186240	2.58	Naphthalene	91
30	9.475	57304	0.79	1-ethyl-4-isopropylbenzene	86
31	9.518	26775	0.37	1,3-diethyl-5-methylbenzene	70
32	9.707	14106	0.20	1,2,3,4,5-pentamethylbenzene	85

33	10.384	13583	0.19	1,2-dimethyltricyclo[3.3.0.02,6]octane	83
34	10.444	4256	0.06	N-(4-methoxyphenyl)decane-1-sulfonamide	82
35	10.544	9196	0.13	2-(hydroxyimino)-1-phenylbutane-1,3-dione	85
36	11.197	75604	1.05	6-methylheptan-1-ol	79
37	11.369	9128	0.13	2,2-dimethylbutane	89
38	11.462	4016	0.06	N-ethyl-N-tetradecylmethanesulfonamide	74
39	12.554	57001	0.79	1,1-dimethylcyclopentane	88
40	14.986	13787	0.19	1,2,3-Trimethyldiaziridine	87
41	15.708	327436	4.54	tetradecan-1-ol	94
42	15.890	21276	0.29	1,2,3-Trimethyldiaziridine	84
43	17.273	57358	0.79	6-methylheptan-1-ol	89
44	17.464	10137	0.14	4,5-dimethyloctane	87
45	17.682	6032	0.08	1-bromopentaborane(9)	100
46	19.642	6641	0.09	2,2-dimethylbutane	95
47	20.758	69546	0.96	tridec-1-ene	86
48	20.937	88668	1.23	3,3-dimethylhexane	89
49	21.101	6905	0.10	1,2-dineopentyl-1l6,2l4-disulfane-1,1,2-trione	96
50	21.735	15528	0.22	3,5-dimethylhex-1-ene	88
51	22.024	53408	0.74	2,6-Dimethyl-6-trifluoroacetoxyoctane	83
52	23.221	100482	1.39	3,7-dimethyloctyl 2,2,2-trifluoroacetate	87
53	23.768	35036	0.49	1-iodononane	89
54	24.096	157419	2.18	3,7,11-trimethyldodecan-1-ol	86
55	24.195	49643	0.69	8-methyldec-1-ene	87
56	24.353	487613	6.76	Cetene	88
57	24.696	468606	6.49	(7R,11R,E)-3,7,11,15-tetramethylhexadec-2-ene	92
58	25.285	39295	0.54	2-(4-fluorophenyl)-2-oxoethyl 3-(1H-tetrazol-1-yl)propanoate	79
59	25.618	19239	0.27	Heptanonitrile	80
60	25.875	7481	0.10	4-Methyl-3-methylene-2-oxetanone	86

61	26.186	21877	0.30	Histamine	81
62	28.245	4861	0.07	(Z)-but-2-en-1-ol	100
63	32.894	30757	0.43	2-ethoxyethyl (2-methoxyethyl) carbonate	93
64	33.647	30143	0.42	Octane-4,5-diol	100
65	34.569	21118	0.29	(5R,6S)-decane-5,6-diol	100
66	37.584	12581	0.17	Di(heptan-3-yl) glutarate	79
67	38.141	6744	0.09	Octane-4,5-diol	100
68	39.503	14906	0.21	tridec-1-yn-4-ol	80
69	39.766	21007	0.29	1,8-cis-Undecadien-5-yne 3,7-bis-trimethylsilyl ether	78
70	43.401	15933	0.22	tridec-1-yn-4-ol	80

Solid phase characterization

Composition of the solid phase of HTL, in terms of CNH (Table S8).

Table S8. Elemental composition (CHN) of the generated solid phase.

Experimental condition			C (%)	H (%)	N (%)
Temperature (°C)	Time (min)	Catalyst (%)			
270	30	0	27,07	4,51	2,04
270	120	0	24,89	3,88	2,12
270	75	10	23,71	3,71	1,99
270	30	20	19,95	3,05	1,64
270	120	20	21,34	3,18	1,93
320	75	0	15,84	2,67	1,85
320	30	10	15,22	2,56	1,69
320	75	10	17,71	2,74	1,82
320	75	10	14,23	2,78	1,67
320	75	10	11,73	2,10	1,41
320	120	10	16,72	2,68	1,90
320	75	20	20,63	3,21	1,94
370	120	0	22,99	3,08	1,71
370	30	0	19,05	3,12	2,17
370	75	10	14,28	2,07	1,44
370	30	20	13,21	1,80	1,34
370	120	20	16,35	1,76	1,49
